# Exploring factors that influence COVID-19 vaccination intention in China: Media use preference, knowledge level and risk perception

**DOI:** 10.3389/fpsyg.2022.954073

**Published:** 2022-09-12

**Authors:** Xuejiao Chen, Yuhan Liu, Guoming Yu

**Affiliations:** ^1^Lab of Cognitive Neuroscience and Communication, School of Journalism and Communication, Beijing Normal University, Beijing, China; ^2^State Key Laboratory of Media Convergence Production Technology and Systems, Beijing, China

**Keywords:** COVID-19 vaccine, media use preference, knowledge level, risk perception, vaccination intention

## Abstract

Vaccine is one of the most effective means to deal with the COVID-19 pandemic in many countries, but vaccine hesitancy has been always widespread among people due to individual differences in access to vaccine information. This research aims to empirically investigate the relationship between media use preference (video-based and text-based), knowledge level, risk perception and willingness to vaccinate among Chinese residents. A cross-sectional survey of a Chinese sample (*N* = 885) was carried out to explore factors that influence the COVID-19 vaccination intention of Chinese residents. The empirical results show that the knowledge level and risk perception of Chinese residents positively contribute to vaccination intention. People with video-usage preference have lower levels of knowledge about the COVID-19 vaccine than those with text-usage preference. People’s risk perception of the COVID-19 pandemic is not influenced by their media use preference or knowledge level, as COVID-19 is a global pandemic and a significant social risk. The current study yields health-related implications for the role of media use preference in vaccination intention.

## Introduction

The COVID-19 pandemic is sweeping the world, causing more than 245 million infections and more than 4.98 million deaths by October 2021 (Worldometers, 2021). Vaccine development is hailed as a long-term solution to the prevention and control of global health crises (Marwah et al., 2021). However, it is not a sufficient basis for many individuals to receive the vaccination without protest. Residents have experienced vaccine hesitancy, indicating that people doubt the benefits of vaccines, worry about their safety, question the need for vaccination, and always associate the vaccine with certain diseases (Shelby and Ernst, 2013). The coverage of vaccination has been estimated to probably reach 75–80% when the COVID-19 vaccine is going to stop a pandemic (Bartsch et al., 2020). As a consequence, the unvaccinated population can lead directly to disease outbreaks.

Communication and media environments are considered to be potential drivers of vaccine hesitancy. In China, the communication environment is somewhat different from that in western countries. First, there are more vaccination-promoting messages on Chinese social media and official media. The Chinese government is much stricter in controlling false information about vaccines on social media (Ouyang et al., 2022). Second, Chinese residents’ opinions on vaccines are largely influenced by their trust in media rather than content (Zhang et al., 2022). They prefer to believe in traditional media channels (Wu and Shen, 2021) and are more influenced by official media (Chen et al. Chen et al., 2021). Third, information access channels for Chinese residents vary greatly (Yu et al., 2021).

Existing studies have investigated the relationship between attitudes, intentions, and behaviors of COVID-19 vaccination based on the communication environment in western countries (Chen et al., 2021; Mir et al., 2021; Thaker and Subramanian, 2021). Some focused on how the interaction of attitude and cognition affects willingness to vaccinate (Carcelen et al., 2021; Mir et al., 2021); some focused on how information features affect risk perception and willingness to vaccinate, such as risk information (Ansari-Moghaddam et al., 2021) and misinformation (Thaker and Subramanian, 2021). However, most studies on COVID-19 vaccine hesitancy only examined these factors separately, which ignored the media use preference as a potential factor influencing vaccination intention. In China, there is a divergence in people’s information access channels. Some studies have found that people always use their media repertoires built by different media choices and preferences to form perceptions (Hasebrink and Popp, 2006). In other words, there is not a clear answer to the relationship between media use preference and vaccination intention.

## Literature review and model development

### Theoretical background

Health behavior refers to actions taken by individuals to avoid risky behaviors and lead to health improvement (Weinstein, 1993; Conner and Norman, 2005). Up to now, there has been a considerable body of studies that recognize the critical role played by social cognitive factors in predicting health behavior (Bandura, 1998; Schwarzer and Renner, 2000; Conner and Norman, 2005). Cognition in psychology is generally considered an information-processing pattern of people’s psychological function (Sternberg and Sternberg, 2016). Some scholars defined cognition as a collection of all mental processes and abilities associated with knowledge, memory, perception and even decision-making (Neisser, 1976; von Eckardt, 1995). According to the theory of social cognition proposed by Bandura (1986), cognition, vicariousness, self-reflection and self-regulation play a central role in processing information. He believes that psychosocial functioning can be explained by triadic reciprocal causation. From the transactional perspective of self and society, environmental events, personal factors and behavioral patterns all serve as interacting determinants that influence each other bidirectionally (Bandura, 2001). Most external effects, the environmental factors he conceptualized, influence behavior through cognitive processes. In addition, cognitive factors exert an in-depth influence on which environmental information is observed, what meaning is given to them, and whether they have a lasting impact.

Results from earlier studies suggest a positive association between cognition and behavioral intention (Dunkley et al., 2011; Winter, 2012). In the context of COVID-19 vaccine information and dissemination, the environmental factors that influence behavior refer to the access to COVID-19 vaccine-related information in the media form of video or text. Moreover, the cognitive output refers to individuals’ level of knowledge, that is, to evaluate their ability to correctly identify misinformation and measure the percentage of people’s correct answers and risk perception. This lays the foundation for a contemplation process early in the motivation phase (Schwarzer and Renner, 2000). Behavioral intention factors refer to individuals’ intention to receive the COVID-19 vaccine. In this theoretical framework, three groups of variables were proposed, which contribute to information processing and decision-making, namely, environmental factors, cognitive factors, and behavioral intention factors.

### Media use preference

Communication scholars have been prompted to speculate on the influence of abundance on choice behavior since the number of information and entertainment choices available to media users has rapidly increased in the last decade (Panek, 2016). Users have a wealth of choices and unprecedented control over where, how and when to obtain news according to their preferences (Mangold and Bachl, 2018). Thus, today’s media users may show more different modes of using news compared to earlier work. Previous studies have identified that social media platforms contribute significantly to the production and diffusion of misinformation (Allcott and Gentzkow, 2017; Shin et al., 2018; Apuke and Omar, 2021). In terms of media format, online fake news research related to COVID-19 showed that the combination of video and text accounts for the largest percentage of fake news content, followed by the combination of text and photo (Al-Zaman, 2021).

A growing number of published studies also provide evidence that people of a higher social class use video media such as television less frequently (Mangold and Bachl, 2018). People who play the role of an opinion leader prefer media with high-quality information, such as investigative reporting and news commentary (Shah and Scheufele, 2006). Instead, research has proven that people who have lower political interests but more media options may cut down their news consumption and spend more on entertainment in video channels (Huang and Yang, 2022).

There are also differences in Chinese residents’ access to information about the COVID-19 pandemic. Some tend to use traditional channels such as newspapers and television to get information. Some people prefer to use audio and video approaches to share information in a private circle *via* social media (Tang and Zou, 2020).

Therefore, it is assumed that there are preferences and divergences in people’s media use, showing reliance on a certain type of information forms, such as TikTok (video media) and news apps (text media). The present study does not explore all the media forms, but focuses on the most important media forms—video media use preference and text media use preference.

Based on the above discussion, it can be hypothesized:

*H1*: People’s media use habit has shown an obvious divergence between video-preference and text-preference.

### Media use preference and knowledge level

Existing literature suggests that information forms can influence an individual’s comprehension ability and then knowledge level. Some studies have concluded that video does not improve people’s learning ability. When learning from videos, learners’ preference for videos over texts does not transfer to better comprehension (Caspi et al., 2005). In addition, evidence supports that comprehension cannot be guaranteed by using videos to communicate scientific information (Mayer et al., 2005). This may be because audiovisual media can put people in a passive state of acceptance, unable to make people initiative and creative (Merkt et al., 2011).

Moreover, media use preference can lead to differences in the quality of the information received, which further affects the level of knowledge. Compared with some text-based reports written by mainstream media, there is information overload combined with gate-keeping failures in video media (Garrett, 2011). A study analyzed user-generated videos about the HPV vaccine on YouTube, finding that most of these videos were negative in tone and disapproved of the HPV vaccine (Briones et al., 2012). As media technology empowers each person, any information can be diffused on the Internet, which dissolves the role of traditional gatekeepers, and a large amount of uncensored misinformation enters the channel. People use the information of uneven quality as a basis for decision-making, further increasing the possibility of misleading information dissemination (Miles et al., 2000; Kata, 2010; Pandey et al., 2010).

Based on the above discussion, it can be proposed:

*H2*: Video media use preference is negatively correlated with the knowledge level of vaccine information.

*H3*: Text media use preference is positively correlated with the knowledge level of vaccine information.

### Media use preference and risk perception

People develop risk perception by receiving the corresponding risk information from media channels, so the risk perception of individuals will vary with media use. Previous studies have concluded that media is a risk amplifier (Kasperson et al., 1988). According to the social learning theory proposed by Bandura (1973), people learn through both action and observation. This means that all the experience we have gained, even second-hand, can lead us to learn about the world. So when it comes to the relationship between risk perception and media use, Bandura says that the mimetic environment created by television distorts the real environment and gives us unrealistic fears. This is because the content of many programs is much more serious than in the real world.

In recent years, many studies have found that media channels and information forms have an important impact on risk perception, which is a key factor affecting vaccination intentions (Renn et al., 1992; Brewer et al., 2007; Kahan et al., 2008; Liao et al., 2013). A study compared individuals’ perception of the risk of eating contaminated fish using pamphlets and classroom lectures, showing that those who received information in the form of classroom lectures perceived higher risks than those who read pamphlets (Burger et al., 2003). It implied that scenario-based and visual information forms could increase the level of risk perception. Moreover, many studies on anti-smoking advertising have also found that anti-smoking warnings in the form of pictures or videos are much more effective than those in the form of texts to inspire risk perception (Evans et al., 2016; Nagelhout et al., 2016).

According to the Dual Coding Theory (DCT), the human mind has two types of mental representations, namely, verbal and visual information (Paivio, 1978). Textual information can generally only mobilize individuals’ mental representation of verbal information, while video information can activate these two mental representations. Considering the involvement and participation of information processing, videos are more likely to increase people’s perception of risk through activating visual mental representation.

Based on the above discussion, it can be put forward:

*H4*: Video media use preference is positively associated with risk perception on COVID-19 virus information.

*H5*: Text media use preference is negatively associated with risk perception on COVID-19 virus information.

### Knowledge level, risk perception and vaccination intention

People are always selectively exposed to some media and content in a wealth of information environments, leading to different levels of health knowledge and a series of disease risk perceptions generated in media and information environments.

In terms of vaccination intention, studies have concluded that it is important to disseminate information about vaccines to increase people’s willingness to vaccinate (Betsch and Wicker, 2012). Thus, personal health knowledge is an important basis for health-related behaviors (Wood et al., 1985). Those with lower levels of knowledge are more likely to associate vaccines with negative events and doubt the safety of vaccines, which may reduce their willingness to get vaccinated (Reyna, 2012; Zheng et al., 2021). A survey reported that 89.2% of health care workers with a high level of vaccine knowledge chose to get vaccinated in the first place after the vaccine became available in China (Li, 2021).

Researchers mentioned that risk perception is a non-negligible predictor. For example, Zhang et al. (2011) found that the possibility of spreading influenza to patients, the mortality risk of H1N1, the vulnerability of people to influenza or H1N1 and other risk perception items were predictors of vaccination. Schwartz et al. (1995) found that the overestimation of ovarian cancer risk can lead women to take positive actions to cope with the disease, such as self-learning and seeking medical treatment. Yaqub et al. (2014) reviewed 34 research articles by meta-analysis, including 15,988 subjects, finding that public vaccination behavior can be significantly predicted by the effect sizes (es) of risk perception.

It is also found that there is a correlation between individual knowledge level of pandemic diseases and perceived risk. Knowledge is often used as an explanatory variable for public attitudes, with an implicit subtext that knowledge can be used as a proxy variable for cognitive ability. The Accessibility/Diagnosticity Theory suggests that there are different information-processing strategies existing between consumers with high-and low-knowledge information. When people are in ambiguous situations, it will be difficult to make judgments, which increases the perceived decision risk (Chiou et al., 2002). More precisely, people with higher levels of knowledge about a particular vaccine are more likely to be aware of the consequences of pandemic and therefore they will perceive a higher risk of environment (Zhang et al., 2011). Conversely, lack of knowledge can interfere with people’s ability to extract the basic meaning or gist of information, which may reduce the perceived risk of pandemic (Reyna, 2012; Rozbroj et al., 2019). In addition, Pew Internet data also showed that 75–80% of users seek health information online (Pew Internet & American Life Project, 2008), indicating that improving the level of knowledge through information-seeking behavior is an effective way to deal with the anxiety of risks. Zhong et al., (2021) found that risk perception can be influenced by knowledge of the disease in a growing pandemic.

In summary, people’s willingness to get vaccinated is the result of the combined effect of knowledge level and risk perception. However, existing studies have been conducted in specific contexts, including SARS (Brug et al., 2004; Smith, 2006) and Ebola (Sell et al., 2017). There is a lack of relevant data on the COVID-19 vaccine currently. Therefore, this study aims to provide new data evidence in the context of the COVID-19 vaccine.

Based on the above discussion, it can be put forward:

*H6*: Knowledge level is positively correlated with risk perception.

*H7*: Risk perception is positively correlated with vaccination intention.

*H8*: Knowledge level is positively correlated with vaccination intention.

**Figure 1:**
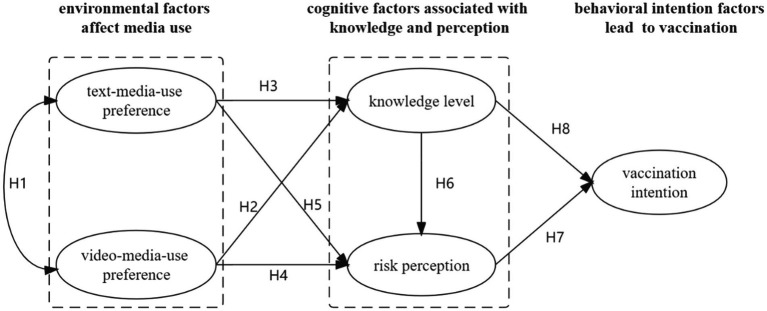
The research model.

On this basis, the research model is proposed, as shown in Figure 1.

## Research method

For the purpose of verifying our framework, empirical research was carried out to test the proposed hypotheses. First of all, a questionnaire was designed for the Chinese residents on the basis of the previous literature and the extant research context. Secondly, we conducted an online investigation by using the Tencent questionnaire platform. Finally, when both the investigation reliability and validity were validated, the got data was explored by using the Structured Equation Modeling through the Amos23.0 and SPSS26.0 tools.

### Measures

To build our research, multi-item scales were produced in accordance with the prior literature. In addition, the survey questionnaire sought data related to the media use preference, knowledge about the COVID-19 vaccine, perception of the risk of the COVID-19 virus and vaccination intention, as well as demographic information.

#### Media use preference

Based on a survey of the media use report of Chinese residents, the top 10 Apps were selected from a total of 50 Apps that Chinese residents use most frequently to obtain information (Yu et al., 2021), including 5 textual media products (i.e., WeChat subscription, Weibo, Zhihu, New Clients, Baidu, and Jinri Toutiao) and 5 video media products (i.e., TikTok, Kwai, Bilibili, Huoshan video, and Xigua video). Besides, by using seven points Likert scale (1 = never, 7 = very often), respondents were asked to select the frequency that they used each of these Apps to obtain the COVID-19 vaccine-related information.

#### Knowledge level

It referred to the total number of right answers to the 12 items. The questionnaire was adapted from the articles on rumors and truths about the COVID-19 vaccine published by the Chinese Center for Disease Control and Prevention in Guangdong (2021). In addition, a total of 12 questions were selected, within which 6 are true and 6 are false. All of these questions served as the measure items to evaluate individuals’ level of knowledge of the COVID-19 vaccine, and the obtained minimum and maximum scores from the scale were 0 and 12, respectively, (in which the correct answer = 1 and the incorrect or unsure answer = 0). Among the, a higher score indicates a higher level of knowledge.

#### Risk perception

The questionnaire was adapted from the scale of Rosenstock (1974). By using seven points Likert scale (1 = strongly disagree, 7 = strongly agree), five items assessed people’s perception of the risk of the COVID-19 pandemic. The risk perception degree was counted as the average value ticked for every item to obtain the risk perception score ranging from 1 to 7.

#### Vaccination intention

The questionnaire was adapted from the well-established research scales of Chien (2011) and Nan and Madden (2012), containing 3 items that assessed people’s vaccination intention by using the 7-point Likert scales (1 = strongly disagree, 7 = strongly agree). Besides, the vaccination intention degree was counted as the average value ticked for every item to generate the risk perception score ranging from 1 to 7.

### Data collection

Through random sampling of Chinese residents, an electronic questionnaire link was posted to people to invite them to attend the online survey. The sampling process was conducted from September 15, 2020, to November 10, 2020. A total of 885 responses have returned, and 885 valid responses remained when the incomplete responses were eliminated. Table 1 shows the respondents’ demographics.

**Table 1:** Demographic information (*N* = 885).

Variable	Category	Numbers	Percentage
Gender	Male	379	42.8
	Female	506	57.2
Area	Urban	514	58.1
	Rural	371	41.9
Marriage	Married	200	22.6
	Unmarried	685	77.4
Age	<16	10	1.1
	16–24	583	65.9
	25–34	223	25.2
	35–50	58	6.6
	>50	11	1.2
Education	High school and below	222	25.1
	Post-secondary	275	31.1
	College and above	388	43.8
Income	<1,000 *yuan*	224	25.3
	1,000–3,000 *yuan*	223	25.2
	3,000–5,000 *yuan*	220	24.8
	5,000–10,000 *yuan*	174	19.7
	>10,000 *yuan*	44	5

## Data analysis and results

### Reliability and validity

Table 2 examines the convergent validity of constructs in our study. The Cronbach’s alpha of the concerned factors had a range between 0.779 and 0.873, which surpassed the 0.6 threshold (van Griethuijsen et al., 2014). The composite reliability (CR) of latent variables was from 0.784 to 0.876, all more than 0.6, which was the suggested critical value suggested by Ryu (2014). As a result, the results pointed out that there was not only a good internal consistency but also a satisfactory reliability level. The convergent validity was verified through the examination of not only the average variance extracted (AVE) but also the standardized factor loadings. Within Table 2, it could be seen that the majority of AVE values were higher compared to the recommended 0.5 threshold (Fornell and Larcker, 1981). In Table 3, the results of the correct identification of knowledge level were indicated.

**Table 2:** Results of confirmatory factor analysis.

Constructs and Items	Factor loadings
**Text-media-use preference (Cronbach’s *α* = 0.779, AVE = 0.440, CR = 0.784)**	
TEX1.1: Wechat	0.32
TEX1.2: Zhihu	0.70
TEX1.3: News Clients (including Tencent News, NetEase News, and so on)	0.84
TEX1.4: Baidu	0.58
TEX1.5: JinRi TouTiao	0.75
**Video-media-use preference (Cronbach’s *α* = 0.837, AVE = 0.557, CR = 0.854)**	
VID2.1: TikTok	0.42
VID2.2: Kwai	0.66
VID2.3: Bilibili	0.65
VID2.4: Huoshan video	0.95
VID2.5: Xigua video	0.92
**Risk perception (Cronbach’s *α* = 0.873, AVE = 0.588, CR = 0.876)**	
RIS4.1: Living and working with people every day increases the likelihood of contracting the COVID-19 virus.	0.70
RIS4.2: Only people over 65 years can be infected with the COVID-19 virus.	0.87
RIS4.3: I have a high probability of contracting the COVID-19 virus.	0.79
RIS4.4: Healthy people can also be infected with the COVID-19 virus.	0.78
RIS4.5: I am worried that I will be infected with the COVID-19 virus.	0.68
**Vaccination intention (Cronbach’s *α* = 0.868, AVE = 0.700, CR = 0.875)**	
INT5.1: I am willing to vaccination once a year in the future if needed.	0.80
INT5.2: If faced with a choice, I would still get vaccinated within a month.	0.86
INT5.3: I will encourage my friends and family to get vaccinated.	0.85

CR, construct reliability; AVE, average variance explained.

**Table 3:** Results of correct identification of knowledge level.

Items	Correctly identified (%)
1. The vaccine is not recommended to be given at the same time as other vaccines for the time being. (T)	74.9
2. Eating a full meal and drinking enough water before the vaccination can avoid adverse reactions. (F)	35.5
3. Nucleic acid test is not necessary before vaccination. (T)	13.7
4. The second dose must be given within 2 weeks to 3 weeks after the first dose. (F)	52.7
5. COVID-19 vaccination is recommended for people 60 years and older because of the health protection it provides. (T)	56.6
6. If in good health, it is recommended that people with chronic diseases also receive the vaccine. (T)	54.0
7. Cancer patients cannot receive the vaccine yet not because of the vaccine itself, but because of a lack of clinical data. (T)	49.8
8. People who work or study in medium or high risk countries or regions serve as a priority group for vaccination. (T)	79.2
9. COVID-19 vaccine may cause cancer. (F)	78.4
10. COVID-19 virus keeps mutating, so the vaccine is useless. (F)	78.8
11. COVID-19 vaccine can change human genes and make people genetically modified. (F)	85.6
12. You can take off the mask after the vaccination. (F)	90.4

The knowledgeable level referred to the total number of correct answers to the 12 items. It ranges from 0 to 12, with 12 indicating the highest level of knowledge of the COVID-19 vaccine and 0 indicating the lowest.

### Model fit assessment

Generally speaking, the data offers a good model fit, which could be confirmed through the estimation of several model fit test statistics: comparative fit index (CFI) = 0.929, the standard root mean square residual (SRMR) = 0.06, *χ*^2^ = (144, *N* = 885) = 743.249, *p* < 0.001, the goodness-of-fit indices (GFI) = 0.914, adjusted goodness of fit index (AGFI) = 0.887, and the root mean square error of approximation (RMSEA) = 0.069. All of the results point out an excellent absolute match of the model.

### Hypothesis testing

**Figure 2:**
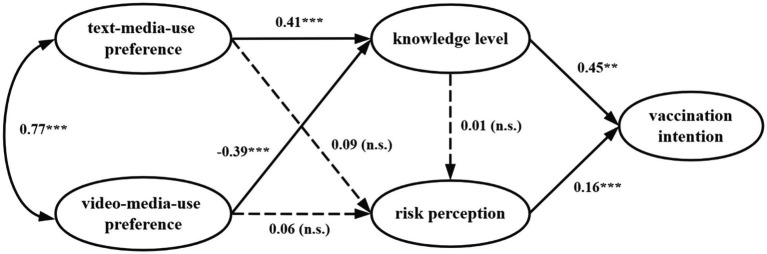
Results of the research model. *p < 0.05, **p < 0.01, ***p < 0.001, n.s.: non-significant at 0.05.

H1 to H8 were all tested through the examination of path coefficients between the different variables. Figure 2 shows the results.

In terms of accessing information about the COVID-19 vaccine, there is a significant correlation in people’s media use preference between video-based and text-based (*β* = 0.77, *p* < 0.001), which supported H1. It was found that the preference for using video media to obtain information exerted a negative impact on people’s knowledge level (*β* = −0.39, *p* < 0.001), and the preference for using text-based media to obtain information exerted a positive impact on people’s knowledge level (*β* = 0.41, *p* < 0.001), supporting H2 and H3. However, H4, H5, and H6 were not supported since preference for using text-based media, preference for using video-based media and knowledge level was not associated with people’s risk perception. Risk perception significantly and positively impacts vaccination intention (*β* = 0.16, *p* < 0.001), supporting H7. In addition, it was also verified that knowledge level had a positive and significant linkage to vaccination intention (*β* = −0.45, *p* < 0.01), thereby supporting H8.

## Discussion and conclusion

Our hypotheses were verified by the empirical research results.

First of all, the findings showed that a significant positive correlation existed between the video-media-use preference and the text-media-use preference, thereby implying that people who frequently use textual media also use video media to access COVID-19 vaccine information. We assumed that citizens’ media exposure diverges into two different camps, which were video-based and text-based, but the results of the study are contrary to our hypothesis. This might be a media repertoire that was created because media users combined different media contacts into one comprehensive contact pattern (Hasebrink and Popp, 2006). This suggested that people combined contacts with different media and different types of content instead of obtaining information from only one media channel. Furthermore, although not consistent with our hypothesis, the result of the study was in line with the previous studies. Taneja et al. (2012) found that there are more than three media repertoires powerfully tied to the rhythms of people’s daily lives. Due to the selective media use, the media repertoires also cause multimethod approaches in converging media environments.

Secondly, we found that compared to individuals with video-media-use preferences, individuals with text-media-use preferences have significantly higher levels of knowledge about vaccines, hence supporting the static media hypothesis. Compared to video information, static text reduced extraneous processing and facilitated the processing of critical information (Mayer et al., 2005). According to Merkt et al. (2011), audiovisual media can not only leave people in a passive state of acceptance but also reduce their creativity. This confirmed the view of this study, which manifested that individuals might not think seriously about the meaning of the content when they received COVID-19 vaccine information through video channels, but the opposite is true for individuals who receive information through textual media. In addition, media use preference could reflect social class characteristics to some extent. To be specific, some people with higher comprehension tended to rely on textual media, while some people with lower comprehension tended to rely more on video media. Taking into account that the differences in information reception channels could further create cognitive and emotional stratification, it was implied that those who prefer text-based media are more capable to interpret vaccine information, while those who prefer video-based media are more easily misled by narrative and emotional fake news.

Thirdly, it was found that people’s risk perception and knowledge level had a significant and positive linkage to vaccination intention. H7 confirmed a positive correlation between risk perception and vaccination intention, which was consistent with the result of the previous studies that risk perception served as an important factor influencing the public’s behavior. People with higher risk perception of the pandemic are more likely to be vaccinated as their acceptance of COVID-19 was associated with the risk perception degree (Zeballos Rivas et al., 2021). In addition, the establishment of H8 suggested that they tended to make more positive vaccination decisions as the public becomes more knowledgeable about social risk events. This finding demonstrated that there were knowledge gaps between different groups. Risk communicators need to be more aware of the knowledge needs of vulnerable groups, which also conformed to the research results of other scholars (Zhong et al., 2021). Interestingly, in this study, people’s perceived risk of the COVID-19 virus was taken as a research variable, but the study by Zheng et al. (2021) used the perceived risk of COVID-19 itself as a variable, and they also confirmed that people’s knowledge level had no direct association with vaccination intentions, whereas was positively and indirectly related to vaccination intentions by reducing the perception of vaccine side effects. Thus, our study extends their established findings and reveals new factors influencing people’s willingness to vaccinate.

In summary, we confirmed in this study that when the public had more comprehensive knowledge about COVID-19 and a higher perception of risk in their social environment, they tended to be more willing to make vaccination decisions consistent with their health status.

However, our several propositions have not been confirmed following the results of the current data analysis.

Firstly, results demonstrated that media use preference and individuals’ knowledge level did not affect their risk perception of COVID-19, which did not conform to our hypothesis. There was a widespread belief argues that the media represents the sources of vital importance for people’s risk perception (Bastide et al., 1989). How and how much influence was another question even if we considered that media did influence people’s risk perceptions. In addition, we found no difference in risk perception between individuals with video-media-use preference and those with text-media-use preference, which was also opposed to the finding of Anderson et al. (2013), that is, the more comprehensive and vivid the media information, the weaker the individual’s risk perception. It was logical that as a *Hot medium* that reduces the cognitive load, video (compared to text alone) should evoke a stronger perception of the risk of COVID-19. However, this is not supported by our findings. This might be because their study focused on the impact of emerging technology (like nanotechnology) on individual risk perceptions, while our findings confirmed that as COVID-19 is a global pandemic, people’s perceptions of significant social risk events do not change depending on their media exposure preferences or knowledge level.

In other words, a general perception had been formed in people’s minds when the media broadcasted to the whole society that “COVID-19 is dangerous!” in China. This perception did not vary according to the media forms. In addition, people’s risk perceptions depended heavily on their pre-existing views instead of being always influenced by media channels. Thus, individuals with a preference for either text or video media did not witness a significant difference in their perceived risk of COVID-19. This was consistent with the previous study that compared to other potential health threats, people had a higher level of risk perception for COVID-19, which was not altered by the habits of media use preference.

Secondly, as the previous discussion suggests, people’s risk perception of the pandemic may not simply be influenced by media use preferences. Risk perception is a multidimensional construct (Wilson et al., 2019). Therefore, in our study, we further proposed H6 from the perspective of cognitive factors to clarify the impact of people’s level of vaccine knowledge on the perceived risk of COVID-19 pandemic, but the results did not support our hypothesis. This may be because that this study defines and measures knowledge level in the context of objective knowledge, rather than subjective knowledge, which includes the perceptions of vaccine effectiveness, safety, and importance. Researchers have proposed that there are two categories of knowledge used in information consumers research, which are (a) subjective knowledge (or perceived knowledge), referring to the individual’s perception of how much she/he knows; (b) objective knowledge (or measured knowledge), which is defined as a measure what an individual knows (Raju et al., 1995).

In this study, the objective knowledge we measured consisted mainly of the identification of COVID-19 vaccine rumors. Subjective knowledge, on the other hand, includes perceptions of vaccine effectiveness, importance, and safety. The level of people’s subjective knowledge about vaccines is another issue regarding trust in vaccines, which may be an alternative interpretation of factors that play a role in the risk perception. In other words, people with higher levels of subjective knowledge about a particular vaccine may have a better understanding of its potential importance and effectiveness. This will strengthen their trust in vaccination and therefore they will perceive the environment as less risky (MacDonald et al., 2012; Liu and Yang, 2021). Future vaccine promotion initiatives should take the role of subjective knowledge of vaccine into account when addressing the negative consequences of risk perceptions of the pandemic.

In conclusion, understanding the factors that influence COVID-19 vaccination intention is a critical step in vaccine promotion initiatives. This study proposes a three-domain model: environmental factors that affect media use preference (video-based vs. text-based), cognitive factors that associated with knowledge level and risk perception, and behavioral intention factors that lead to vaccination. The empirical results show that risk perception was a positive predictor of COVID-19 vaccination intention. People’s media use preference had an indirect effect on vaccination intention through high knowledge level, with video-based media use preference having lower level of knowledge and text-based media use preference having higher level of knowledge.

## Limitations and future directions

Because of the chosen samples and the research context, some of the limitations are likely to impact the generalizability of the findings. First of all, the present study used an online platform to distribute the questionnaires during the survey, and thus the sample selection was inevitably limited to those having a chance to access the Internet, resulting in a large number of respondents in this study being young people. In this case, this group can only be researched at first, and this study can be considered a primary study focusing on this topic.

Secondly, when investigating the respondents’ media exposure channels, we did not assess the quality of the information content they received. This is because the information quality can also influence the public’s willingness to act and is likely to have a negative impact. As a result, future research can make some efforts to use a content quality perspective to explore the impact of media use preferences on the public’s knowledge level and risk perceptions, to identify elements that influence the public’s vaccination intention.

## Data availability statement

The raw data supporting the conclusions of this article will be made available by the authors, without undue reservation.

## Author contributions

XC, YL, and GY participated in the design of the study. XC performed questionnaire design, data analysis, and wrote the manuscript and edited. YL performed data collection and paper revision. GY contributed to the conceptualization of the manuscript, designed the questionnaire, and provided the supervision of the whole research and reviewed the content of manuscript. All authors contributed to the article and approved the submitted version.

## Funding

This manuscript was funded by the State Key Laboratory of Media Convergence Production Technology and Systems (No. SKLMCPTS202103015 and SKLMCPTS202103014) and National Social Science Fund of China (No. 17CXW039).

## Conflict of interest

The authors declare that the research was conducted in the absence of any commercial or financial relationships that could be construed as a potential conflict of interest.

## Publisher’s note

All claims expressed in this article are solely those of the authors and do not necessarily represent those of their affiliated organizations, or those of the publisher, the editors and the reviewers. Any product that may be evaluated in this article, or claim that may be made by its manufacturer, is not guaranteed or endorsed by the publisher.
